# Rapid and specific detection of *Trichophyton rubrum* and *Trichophyton mentagrophytes* using a loop-mediated isothermal amplification assay

**DOI:** 10.1016/j.mex.2022.101891

**Published:** 2022-10-27

**Authors:** Mi-Ran Seo, Hyo Seok Kim, Young Bok Lee, Sun Shin, Yeun-Jun Chung

**Affiliations:** a ConnectaGen, Hanam 12918, Korea; b Department of Dermatology, Uijeongbu St. Mary's Hospital, The Catholic University of Korea, Uijeongbu, Korea; c Department of Biomedicine & Health Sciences; d Department of Microbiology; e Precision Medicine Research Center; f IRCGP, The Catholic University of Korea, Seoul 06591, Republic of Korea

**Keywords:** onychomycosis, Trichophyton rubrum, Trichophyton mentagrophytes, loop-mediated isothermal amplification

## Abstract

*Trichophyton rubrum* and *Trichophyton mentagrophytes* are the main causative pathogens of onychomycosis. However, the sensitivity and specificity of conventional microscopic tests are insufficient for reliable diagnoses of onychomycosis. In this study, we developed loop-mediated isothermal amplification (LAMP) assays for the rapid and specific identification of the two major *Trichophytons* spp. We designed LAMP primers targeting the internal transcribed spacer 1 region of the *T. rubrum* and *T. mentagrophytes*. Through rigorous optimization of the reaction conditions, we defined a universal reaction condition for both LAMP assays.

## Highlights

• Under these conditions, the detection limits were 5 pg for *T. rubrum* and 500 fg for *T. mentagrophytes*, without any non-specific signals from eight non-*Trichophyton* fungal spp*.* Regarding the reaction time, a positive signal was detected within 30 min of the start of the reaction.
• To verify whether our LAMP systems were applicable to onychomycosis clinical samples, 19 microscopic observation-positive toenail samples were tested. The sensitivity and specificity of our LAMP assays were 100% (95% confidence interval [CI] 76.8–100% and 47.8–100%) and 100% (95% CI 15.8–100% and 80.5–100%), respectively.
• Taken together, the developed LAMP assays can be useful for detecting *T. rubrum* and *T. mentagrophytes* in outpatient settings and point-of-care testing*.*

## Graphical abstract

**Figure:**
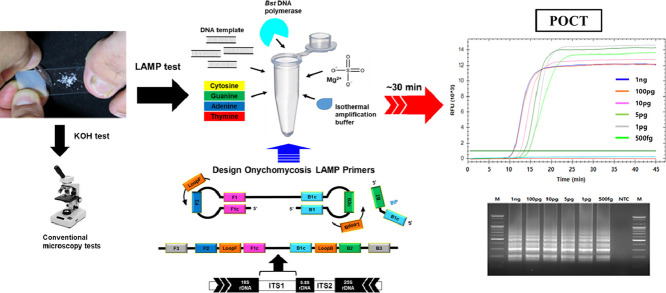

Specifications Table

**Table:** 

Subject area:	Immunology and Microbiology
More specific subject area:	Mycology
Name of your method:	LAMP assays for Trichophyton rubrum and Trichophyton mentagrophytes
Name and reference of original method:	https://doi.org/10.1093/nar/28.12.e63
Resource availability:	Yes

**Method details**

## Introduction

Onychomycosis, the most common nail disease, accounts for ∼50% of all nail disease cases, in which nails are invaded by fungi, such as dermatophytes, yeasts, and non-dermatophytes [1], [2], [3], [4]. In addition to nail problems such as nail discoloration and thickening, onychomycosis can affect daily activities due to pain and discomfort. As onychomycosis is difficult to treat and often becomes chronic with repeated relapses, it is important to identify the causal organism and commence treatment properly [3], [4], [5]. Although general hygiene has improved, the prevalence of onychomycosis is increasing in Korea owing to several factors, including increase in the elderly population, high prevalence of diabetes, and increased use of immunosuppressants [3,4,[6], [7], [8]]. Pathogenic dermatophytes are a group of fungi that can invade keratinized tissues, such as the skin, hair, and nails. More than 40 types of dermatophytes have been identified, and among them, *Trichophyton rubrum* is the most prevalent species, followed by *Trichophyton mentagrophytes*, worldwide, including in Korea [8,9].

The standard method for onychomycosis diagnosis is microscopic observation of the fungus treated with KOH and identification of the fungal species by fungal culture [10]. Microscopic observation is simple and fast, but its sensitivity and specificity are low [11], [12], [13], [14]. In the case of fungal culture, it takes several weeks, and the possibility of culture failure is high. Recently, several polymerase chain reaction (PCR)-based diagnostic methods have been developed for the rapid and accurate detection of dermatophytes, such as PCR-restriction fragment length polymorphism (PCR-RFLP) [15,16], real-time PCR [17,18], and nested-PCR [19]. However, such PCR-based methods require sophisticated machines and several hours to obtain the result, which is not suitable in outpatient or point-of-care testing (POCT) settings.

Loop-mediated isothermal amplification (LAMP), an approach developed by Notomi et al. in 2000, can amplify DNA or RNA targets without thermal cycling [20]. The LAMP reaction requires up to six target-specific primers. Unlike PCR, LAMP requires strand displacement using *Bst* DNA polymerase, which enables highly sensitive, quick, and efficient amplification under isothermal conditions [20]. In addition, LAMP reaction products can be read through turbidity or fluorescence [20], [21], [22], making the reaction equipment required simpler and smaller than the conventional PCR machines. Owing to these advantages, LAMP technology has been considered ideal for POCT [23]. We, therefore, aimed to develop a LAMP-based rapid and sensitive detection method for the two most common onychomycotic pathogens (*T. rubrum* and *T. mentagrophytes*).

## Method details

### Preparation of fungal genomic DNA

The two positive control strains *T. rubrum* and *T. mentagrophytes* and eight non-*Trichophyton* fungal species (*Epidermophyton floccosum, Microsporum canis, Aspergillus fumigatus, Aspergillus niger, Candida albicans, Candida glabrata, Fusarium solani,* and *Cryptococcus neoformans*) were purchased from the Korean Collection for Type Cultures (KCTC; Jeollabukdo, Korea) and the National Culture Collection for Pathogens (Osong, Korea).Genomic DNA was extracted using a MasterPure Yeast Purification Kit (Epicentre Biotechnologies, Madison, WI, USA) as follows: (i) lysis buffer was added to the samples and vortexed with for 1min; (ii) the treated samples were centrifuged at 13,000 rpm for 10min and supernatant was transferred to a new microcentrifuge tube; (iii) 1 microliter RNase A (100 micrograms/microliter) was added to the supernatant and incubated at 65°C for 15min; (iv) the samples were kept on ice for 5min and MPC protein Precipitation Reagent was added; (v) centrifuged at 13,000 rpm for 10min and supernatant was transferred to a new microcentrifuge tube; (vi) isopropanol was added and centrifuged at 13,000 rpm for 10 min; (vii) the pellet was washed with 70% ethanol; (viii) the nucleic acids were finally eluted with 50 µ*l* of TE buffer. The concentration was determined using NanoDrop 1000 (Thermo Fisher Scientific Inc., Waltham, MA, USA). The extracted genomic DNA was used as a template for LAMP. The internal transcribed spacer 1 (ITS1) region of the rDNA of *T. rubrum* and *T. mentagrophytes* was PCR-amplified and ligated into RBC T&A cloning vectors (Real Biotech Corporation, Banqiao, Taiwan).

### Primer design for LAMP

**Fig. 1:**
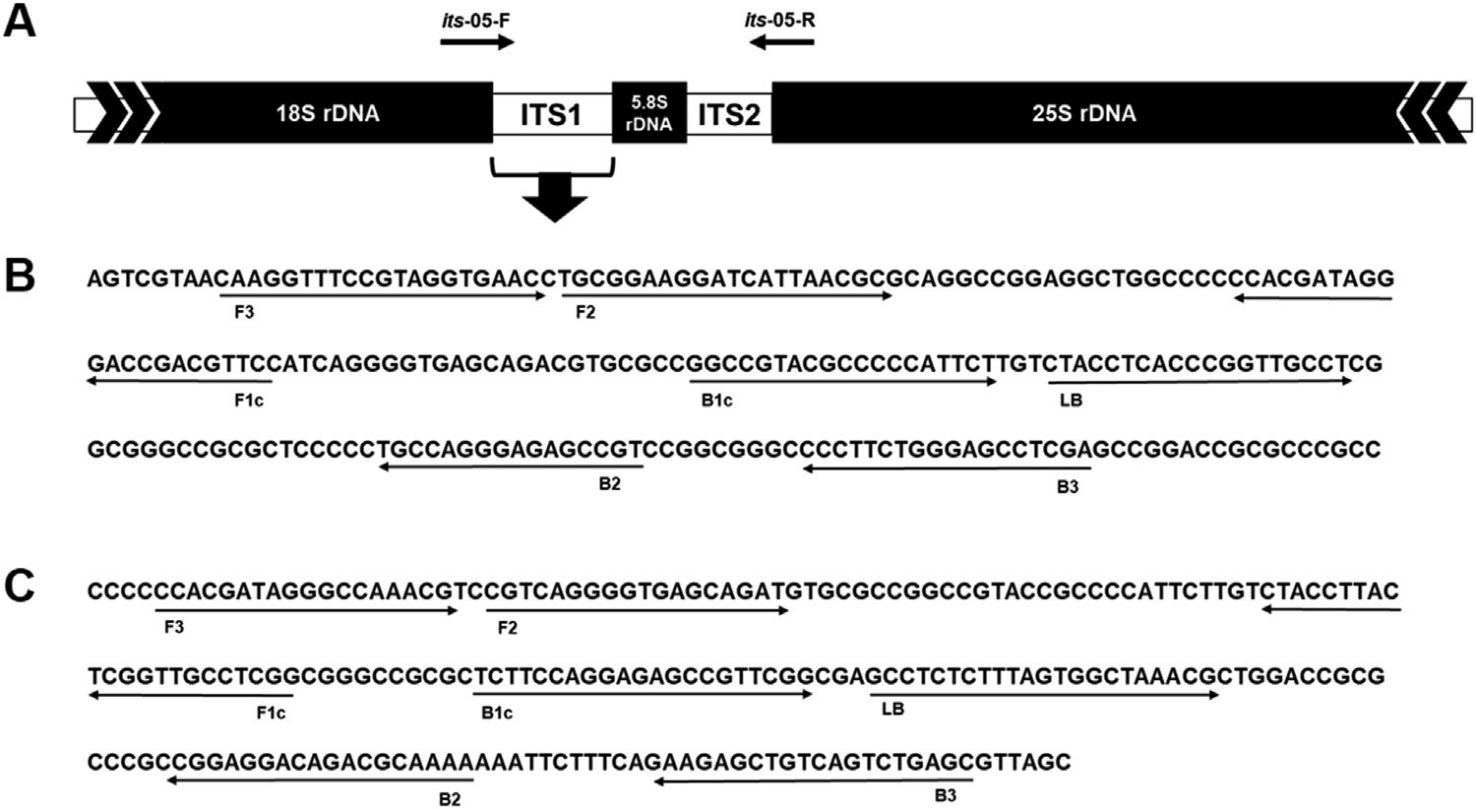
Structure of internal transcribed spacer (ITS) region and design of loop-mediated isothermal amplification (LAMP) primers. (A) Verification strategy of the fungi isolated from the onychomycosis patient samples via PCR-sequencing, as described elsewhere [25]. LAMP primers designed for the detection of *Trichophyton rubrum* (B) and *Trichophyton mentagrophytes* (C). Positions of five primers (F3, B3, FIP [F1c-F2], BIP [B1c-B2], and LB) aligned on the nucleotide sequence of the ITS1 region (GenBank accession Nos.: JX431933 and MH791418, respectively).

The ITS1 regions of *T. rubrum* (GenBank accession No.: JX431933) and *T. mentagrophytes* (GenBank accession No.: MH791418) were selected using BioEdit (version 7.2; https://bioedit.software.informer.com/7.2/). The LAMP primers were designed using Primer Explorer V5 (http://primerexplorer.jp/elamrep5.0.0/index.html; Eiken Chemical, Tokyo, Japan) and manual curation (Fig. 1 and Table 1). F3 and B3 primers were designed to be positioned outside the inner primer. The FIP and BIP primers included the TTTT spacer (5′-TTTT-3′) sequence as described elsewhere [20]. All sets of primers were validated using basic local alignment search tool (BLAST).

**Table 1:** The sequences of the primers for LAMP assay used in this study

Primer	Length(bp)	Sequences (5’-3’)
*T. rubrum*		
F3	20	CAAGGTTTCCGTAGGTGAAC
B3	17	TCGAGGCTCCCAGAAGG
FIP*	45	GAACGTCGGTCCCTATCGTGGTTTTTGCGGAAGGATCATTAACGC
BIP*	39	GGCCGTACGCCCCCATTCTTTTTACGGCTCTCCCTGGCA
LB	19	CTACCTCACCCGGTTGCCT
*T. mentagrophytes*		
F3	18	CCACGATAGGGCCAAACG
B3	20	GCTCAGACTGACAGCTCTTC
FIP*	44	CCGAGGCAACCGAGTAAGGTAGTTTTCGTCAGGGGTGAGCAGAT
BIP*	43	TCTTCCAGGAGAGCCGTTCGGTTTTTTTTGCGTCTGTCCTCCG
LB	22	GCCTCTCTTTAGTGGCTAAACG

Abbreviations: LAMP, loop-mediated isothermal amplification; F3, outer forward primer; B3, outer backward primer; FIP, forward inner primer; BIP, backward inner primer; LB, backward loop primer

⁎ Each inner primers (FIP and BIP) have two binding regions (F1c+F2 and B1c+B2, respectively) connected by a TTTT spacer

### LAMP assay

The LAMP reaction was carried out as described previously, with some modifications [20,21,23,24]. Briefly, the 20 µ*l* LAMP reaction mixture contained 2 µ*l* of 10X Isothermal Amplification buffer (New England Biolabs, Ipswich, MA, USA), 1.5 mM of each deoxynucleotide triphosphate, 6 mM MgSO_4_, 8U of *Bst* 2.0 DNA polymerase, 1.25 µM of SYTO9 (Thermo Fisher Scientific), and 2 µ*l* of *T. rubrum* (or *T. mentabrophytes* DNA template. The composition of the *T. rubrum* and *T. mentagrophytes* LAMP primer mix included 0.2 µM of the outer primers (F3 and B3), 1.6 µM of the inner primers (FIP and BIP), and 0.4 µM of loop primer (LB). The amplification reaction was performed at 60°C for 45 min and terminated by heating at 80°C for 3 min using a CFX96 Touch Real-Time PCR Detection System (Bio-Rad Laboratories, Hercules, CA, USA). The fluorescence curve was captured using Bio-Rad CFX Manager.

### Optimization of LAMP reaction

To optimize the amplification conditions, LAMP was performed at different reaction conditions: LB primer concentrations (0.2, 0.3, and 0.4 µM), reaction temperatures (60, 63, and 65 °C), *Bst* DNA polymerase units (4, 6, 8, and 10U), dNTPs (1.0, 1.2, 1.4, 1.6, and 1.8 mM), and MgSO_4_ concentrations (4, 6, and 8 mM). Nuclease-free water was used as a negative control.

### Specificity and sensitivity of LAMP assay

To evaluate the detection sensitivity of the LAMP assay, genomic DNA from *T. rubrum* and *T. mentagrophytes* was serially diluted from 1 ng/µ*l*to 100 fg/µ*l* and subjected to LAMP. To evaluate the specificity of our LAMP assays, reactions were performed using DNA from eight non-*Trichophyton* fungal species. All LAMP reactions were repeated thrice.

### Clinical samples subjected to the LAMP assay

Toenail specimens from 19 fungus microscopy test-positive patients, diagnosed by a board-certified dermatologist, were collected from Uijeongbu St. Mary's Hospital (Gyeonggi-do, Korea) between August to November 2021. The study design was approved by the Institutional Review Board of Catholic Medical Center (UIRB-면20210716-002). Fungal DNA was extracted using a QIAamp DNA Mini Kit (Qiagen, Hilden, Germany). The extracted genomic DNA was used as a template for the LAMP assay. Before LAMP, the genomic DNA was amplified using primers designed to amplify the full ITS sequence as described elsewhere [25]: 05-forward primer (5′-GATTGAATGGCTTAGTGAGG-3′) and its_05-reverse primer (5′-TTGTTCGCTATCGGTCTC-3′; Fig. 1A). The PCR products were sequenced via Sanger sequencing, and the sequencing results were evaluated using BLAST (https://blast.ncbi.nlm.nih.gov/Blast.cgi) to determine the closest relatives on the NCBI website (http://www.ncbi.nlm.nih.gov).

## Method validation

### Design and validation of LAMP primer sets

Five LAMP primers targeting the ITS1 region of *T. rubrum* and *T. mentagrophytes* were designed: two inner primers (FIP and BIP), two outer primers (F3 and B3), and one loop primer (LB) (Fig. 1b and 1c). To validate whether the primer sets worked properly, we applied the *T. rubrum* and *T. mentagrophytes* LAMP primer sets to the DNA of reference *T. rubrum* and *T. mentagrophytes* under the conditions described elsewhere (60°C for 45 min) [23,24] and confirmed that both LAMP primer sets could amplify the reference DNA (data not shown).

### Optimization of LAMP assay conditions

**Fig. 2:**
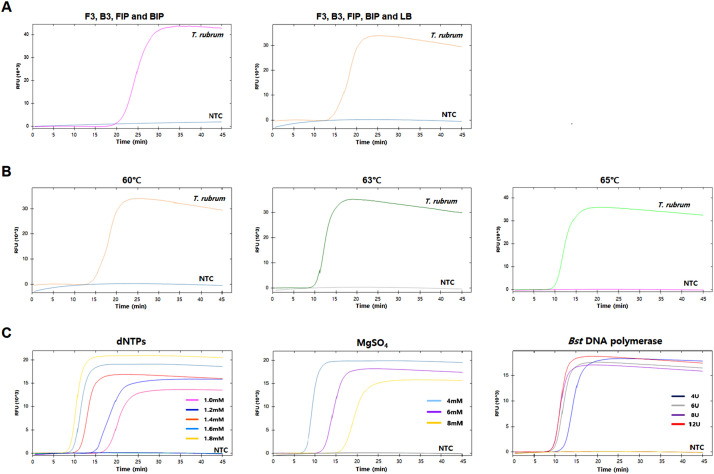
Optimization of *Trichophyton rubrum* loop-mediated isothermal amplification (LAMP) reaction conditions. (A) LAMP result with two different primer sets. (B) LAMP result with three different temperatures. (C) LAMP result with different concentrations of dNTPs, MgSO_4_, and *Bst* DNA polymerase. X-axis, time (min); Y-axis, relative fluorescence unit (10^3^). Nuclease-free water was used as a negative control (NTC).

**Fig. 3:**
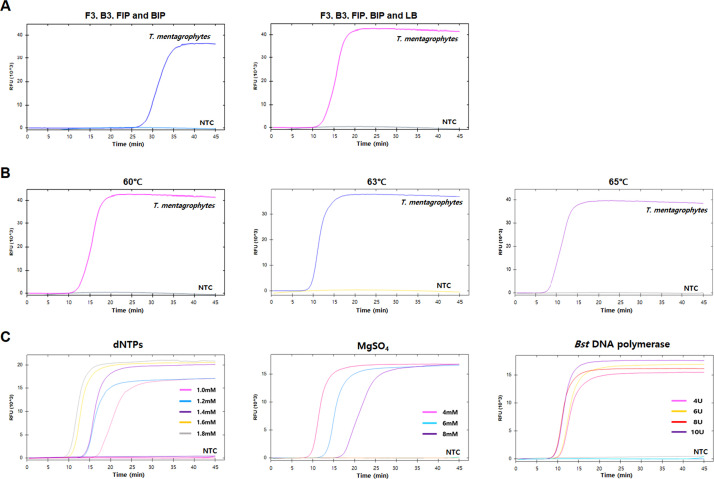
Optimization of *Trichophyton mentagrophytes* loop-mediated isothermal amplification (LAMP) reaction conditions. (A) LAMP result with two different primer sets. (B) LAMP result with three different temperatures. (C) LAMP result with different concentrations of dNTPs, MgSO_4_, and *Bst* DNA polymerase. X-axis, time (min); Y-axis, relative fluorescence unit (10^3^). Nuclease-free water was used as a negative control (NTC).

Next, we attempted to optimize the LAMP reaction conditions. As a first step, we performed the LAMP reaction with two different combinations of primer sets: set 1, where LAMP was performed using two inner and two outer primers without a loop primer; and set 2, where set 1 primers plus LB were used. Set 2 showed faster amplification than set 1 (Fig. 2A and 3A). None of the negative controls showed positive signals during the reaction. Therefore, we decided to include all five primers for both *T. rubrum* and *T. mentagrophytes* LAMP systems. Using primer set 2, we compared three different reaction temperatures (60, 63, and 65°C) and found that 65°C showed the best amplification performance (Fig. 2B and 3B). We also compared different concentrations of dNTPs, MgSO_4_, and *Bst* DNA polymerase. The reaction condition of 1.8 mM dNTPs, 4 mM MgSO_4_, and 8U *Bs*t DNA polymerase showed the fastest amplification without any false-positive signals for both *T. rubrum* and *T. mentagrophytes* (Fig. 2C and 3C). Under these reaction conditions, the amplification signal appeared approximately 20 min after starting the LAMP reaction, without false-positive signals for both targets.

### Sensitivity and specificity of LAMP assays

**Fig. 4:**
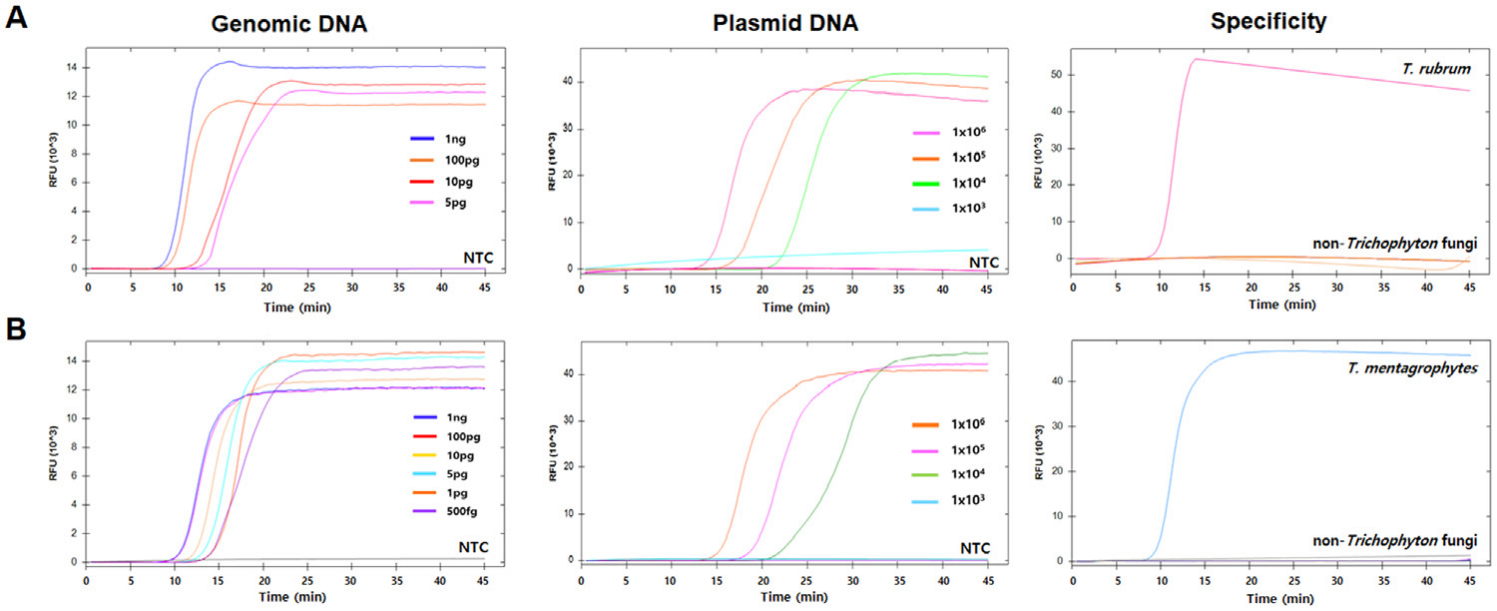
Sensitivity and specificity of *Trichophyton rubrum* (A) and *Trichophyton mentagrophytes* (B) loop-mediated isothermal amplification (LAMP) assays. Serially diluted genomic DNA of *T. rubrum* and *T. mentagrophytes* was subjected to LAMP. Plasmid DNA containing ITS1 region of *T. rubrum* and *T. mentagrophytes* was diluted from 10^6^ copies to 10^1^ copies and subjected to LAMP. To evaluate the specificity, eight non-*Trichophyton* fungi were tested. Nuclease-free water was used as a negative control (NTC).

To test the detection sensitivity of *T. rubrum* and *T. mentagrophytes* LAMP assays, we analyzed the limit of detection (LOD) of each assay using the diluted target DNA to a series of concentrations ranging from 1 ng/µ*l*to 100 fg/µ*l*. The LOD for the *T. rubrum* LAMP ssay was 5 pg of genomic DNA and 10^4^ copies of plasmid DNA per reaction, and that for the *T. mentagrophytes* LAMP assay was 500 fg of genomic DNA and 10^4^ copies of plasmid DNA per reaction (Fig. 4A and 4 B). To evaluate the specificity of the two LAMP assays, we prepared DNA from eight non-*Trichophyton* fungi that could induce tinea and other infectious diseases. Any positive amplification signal was observed from the non-*Trichophyton* fungi DNA, while *T. rubrum* and *T. mentagrophytes* DNA showed specific amplification signals (Fig. 4A and 4B). BLAST analysis revealed that none of the eight non-*Trichophyton* fungi had sequences similar to the *T. rubrum* and *T. mentagrophytes* ITS1 sequence (data not shown).

### Detection of the two Trichophyton species from clinical samples using the developed LAMP assays

To determine whether our LAMP systems can detect *T. rubrum* and *T. mentagrophytes* in clinical samples from onychomycosis patients, we applied the LAMP systems to 19 KOH test-positive toenail samples. To validate the 19 clinical samples, we performed PCR targeting the entire ITS region and performed Sanger sequencing of the PCR products, before conducting the LAMP assay. Of the 19 samples, 17 were ITS PCR-positive, and Sanger sequencing revealed that 14 samples had a *T. rubrum*-specific sequence, two had a *T. mentagrophytes-*specific sequence, and one had a *Malasseziomycetes-*specific sequence. Examples of ITS PCR-sequencing are shown in Supplementary Figure S1. The two other samples were ITS PCR-negative. When we applied the *T. rubrum* and *T. mentagrophytes* LAMP assays to the 19 clinical specimens, 14 were *T. rubrum* LAMP-positive, 2 were *T. mentagrophytes* LAMP-positive, and 3 were *Trichophyton* LAMP-negative (Supplementary Table S1). Taken together, the sensitivity and specificity of the *T. rubrum* and *T. mentagrophytes* LAMP assays were 100% (95% confidence interval [CI] 76.8–100% and 47.8–100%) and 100% (95% CI 15.8–100% and 80.5–100%), respectively.

## Discussion

PCR-based diagnostic methods are commonly used as molecular diagnostic tools in the clinical field worldwide. The high sensitivity of qPCR is a major advantage over conventional diagnostic methods [15,18,25]. However, owing to the running time of several hours, PCR-based methods are not suitable for use in POCT or outpatient settings. In this study, we developed a real-time LAMP assay for the rapid and sensitive detection of *T. rubrum* and *T. mentagrophytes,* the main causative pathogens responsible for 80–90% of onychomycosis cases [[6], [7], [8], [9],^12^,^13^]. LAMP for *T. rubrum* has been reported previously [26]; however, a LAMP assay for *T. mentagrophytes*, another major causal fungus of onychomycosis, was not developed until the present study. This is the first study to develop a diagnostic method for identifying two major causal fungi of onychomycosis.

We designed LAMP primers targeting the ITS1 region of *T. rubrum* and *T. mentagrophytes*. ITS1 is a non-coding region located between 18S and 5.8S ribosomal DNA, where the sequences are strain-specific; therefore, this region can be useful for phylogenetic analysis and identification of fungal subspecies [16,17]. By comparing various reaction conditions, we defined a universal optimal reaction condition for both LAMP assays: isothermal reaction at 65°C, dNTP 1.8 mM, MgSO_4_ 4 mM, and 8U *Bst* DNA polymerase in a 20µ*l* reaction volume. Under these conditions, the LOD obtained for *T. rubrum* (5 pg/reaction) and for *T. mentagrophytes* (0.5 pg/reaction) was more sensitive than that of the LAMP method reported by Watanabe et al. (10 ng/reaction) [26].

We checked whether there was a cross-reaction between *T. rubrum* and *T. mentagrophytes* and found that both targets were detected species-specifically without cross-reaction, supporting the specificity of our LAMP assays. We also tested both assays using eight non-*Trichophyton* pathogenic fungi and observed no positive LAMP signal from the DNA of any of these species, further suggesting the high specificity of our LAMP assays.

To verify whether our LAMP systems are applicable to onychomycosis clinical samples, 19 microscopic observation-positive toenail samples were tested using our LAMP assays. Although the KOH microscopy test is the most popular diagnostic procedure for onychomycosis in the clinical field, it cannot confirm the existence of *T. rubrum* or *T. mentagrophytes*. All the 14 *T. rubrum* and 2 *T. mentagrophytes* confirmed specimens showed specific LAMP-positive results without any cross-reaction. In terms of detection sensitivity, both LAMP systems showed 100% sensitivity. There was no LAMP-positive signal for the other three samples (one *Malassezia* and two ITS PCR-negatives), indicating that the absolute specificity of our LAMP system. Due to limited clinical sample size, the diagnostic efficacy of the LAMP assay could not be established in this study. Further validation study with larger scale clinical samples will be required to establish the diagnostic efficacy. Regarding the process time, positive LAMP signals were detected within 30 min of starting the LAMP reaction in both assays. Considering the time required for DNA extraction and preparation of reaction reagents (∼30 min), the final analysis result can be reported within 1 h of sampling from the patient, which is much shorter than PCR-based detection (which requires ∼3 h) [14].

In this study, two KOH-positive clinical samples were ITS PCR-negative, suggesting the possibility of a false interpretation in the KOH microscopy test. This result is consistent with previous observations indicating the lower sensitivity and specificity of conventional KOH microscopy tests [11], [12], [13], [14]. Another possibility is that the specimens collected for this study did not contain fungi or that the extracted DNA was too sheared due to several reasons, such as inappropriate sample storage or DNA extraction procedure. Although we could not verify this technical possibility with the clinical specimens in this study, our LAMP assays do have superior sensitivity and specificity compared with the conventional KOH microscopy test. Therefore, our LAMP assay could be a useful alternative tool to compensate for the limitations of conventional microscopy tests.

## Conclusion

Our *T. rubrum* and *T. mentagrophytes* LAMP assay could detect both targets with high sensitivity and specificity within 1 h. Considering the time, sensitivity, and specificity, these assays are potentially useful tools for detecting *T. rubrum* and *T. mentagrophytes* in outpatient and POCT settings.

## Declaration of interests

Please tick the appropriate statement below (please do not delete either statement) and declare any financial interests/personal relationships which may affect your work in the box below.

The authors declare that they have no known competing financial interests or personal relationships that could have appeared to influence the work reported in this paper.

Please declare any financial interests/personal relationships which may be considered as potential competing interests here.

## Appendix Supplementary materials

Image, application 1
