# Supplementary material for: Rapid and specific detection of Trichophyton rubrum and Trichophyton mentagrophytes using a loop-mediated isothermal amplification assay
Source: MethodsX. 2022 Oct 27;9:101891. doi: 10.1016/j.mex.2022.101891 (PMC9637954; doi:10.1016/j.mex.2022.101891)
Supplement: Supplementary file 1 [file mmc1.docx]

**Supplementary Materials**

**Supplementary Figure S1.** Verification of the fungi isolated from the onychomycosis patient samples by PCR-sequencing. (A) ITS PCR-sequencing revealed *Trichophyton rubrum* (602bp) specific sequence in 14 clinical samples. (B) ITS PCR-sequencing revealed *Trichophyton* *mentagrophytes* (593bp) specific sequence in 2 clinical samples.


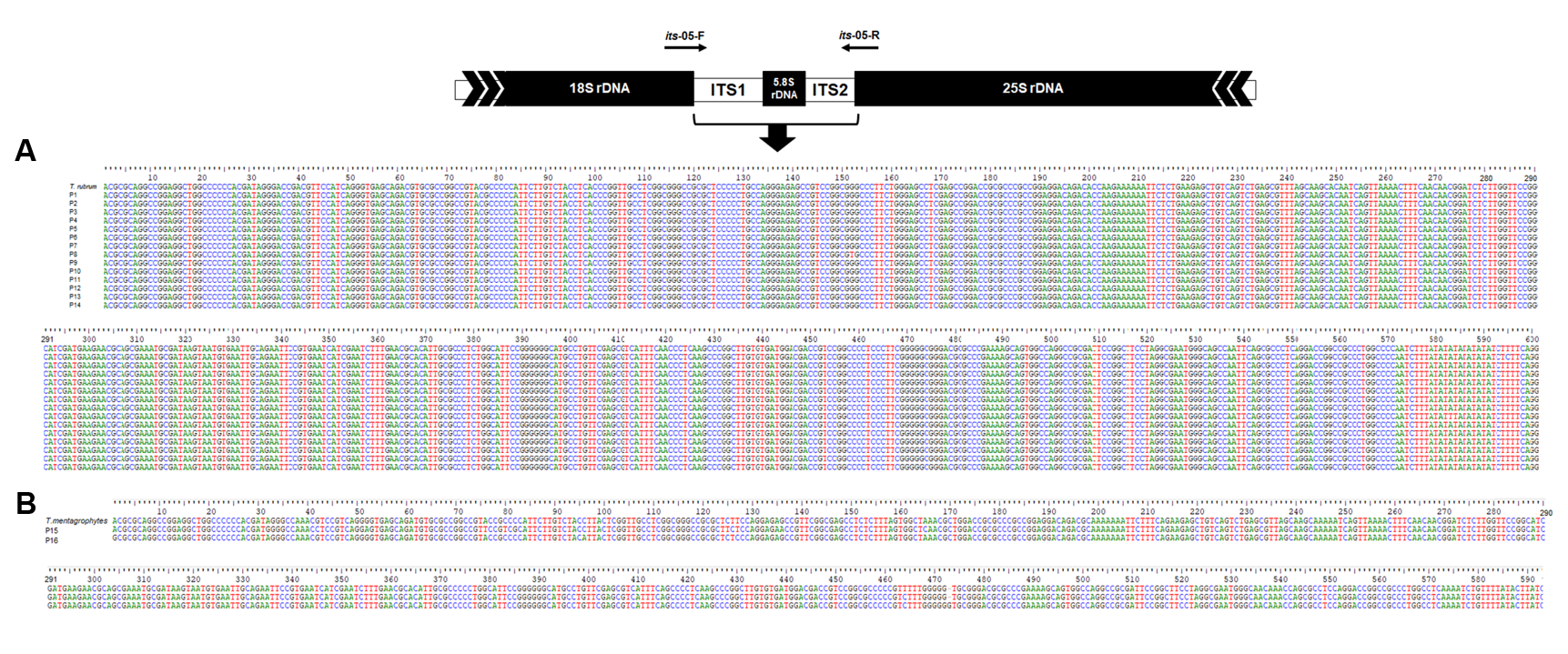


**Supplementary Table S1.** Identification of *Trichophytons* via *T. rubrum* and *T. mentagrophytes* LAMP assays to the 19 clinical specimens.

|  | | LAMP (%) | | | Total |
| --- | --- | --- | --- | --- | --- |
|  |  | *T. rubrum* (+) | *T. mentagrophytes* (+) | (-) |  |
| ITS PCR- sequencing | *T. rubrum* | 14 (73.7) |  |  | 14 |
|  | *T. mentagrophytes* |  | 2 (10.5) |  | 2 |
|  | Others* |  |  | 3 (15.8) | 3 |
| Total | | 14 (73.7) | 2 (10.5) | 3 (15.8) | 19 |

*Others: one had a *Malasseziomycetes*-specific sequence and the other two were ITS PCR- sequencing negative.
